# iGEM and Gene Drives: A Case Study for Governance

**DOI:** 10.1089/hs.2021.0157

**Published:** 2022-02-15

**Authors:** Piers Millett, Tessa Alexanian, Megan J. Palmer, Sam Weiss Evans, Todd Kuiken, Kenneth Oye

**Affiliations:** Piers Millett, PhD, is Vice President for Safety and Security and Tessa Alexanian is a Safety and Security Program Officer; both at iGEM Foundation, Cambridge, MA. Megan J. Palmer, PhD, is a Bio Policy and Leadership Initiatives and Adjunct Professor, Department of Bioengineering, Stanford University, Stanford, CA. Sam Weiss Evans, DPhil, is a Senior Research Fellow, Program on Science, Technology, and Society, Harvard University, Cambridge, MA. Todd Kuiken, PhD, is a Senior Research Scholar, Genetic Engineering and Society Center, North Carolina State University, Raleigh, NC. Kenneth Oye, PhD, is a Professor of Political Science and Director of the Program on Emerging Technologies, Massachusetts Institute of Technology, Cambridge, MA.

**Keywords:** iGEM, Gene drives, Adaptive governance, Risk management, Governance, Adaptive risk management

## Abstract

Gene drives have already challenged governance systems. In this case study, we explore the International Genetically Engineered Machine (iGEM) competition's experiences in gene drive-related research and lessons in developing, revising, and implementing a governance system. iGEM's experiences and lessons are distilled into 6 key insights for future gene drive policy development in the United States: (1) gene drives deserve special attention because of their potential for widescale impact and remaining uncertainty about how to evaluate intergenerational and transboundary risks; (2) an adaptive risk management approach is logical for gene drives because of the rapidly changing technical environment; (3) review by individual technical experts is limited and may fail to incorporate other forms of expertise and, therefore, must be complemented with a range of alternative governance methods; (4) current laboratory biosafety and biosecurity review processes may not capture gene drive research or its components in practice even if they are covered theoretically; (5) risk management for research and development must incorporate discussions of values and broader implications of the work; and (6) a regular technology horizon scanning capacity is needed for the early identification of advances that could pose governance system challenges.

## Introduction

Synthetic RNA-guided gene drives have recently been developed to bias inheritance in microbial, insect, and mammalian populations toward engineered genetic traits.^1-3^ The potential for RNA-guided gene drives to be adapted to new genetic or species targets has drawn excitement around their potential use in controlling invasive species and disease vectors. However, their use in the ecosystem brings major governance challenges, as the potential impacts of gene drives span national boundaries and generations.^4^ Whether a gene drive is an anomaly within the current system of regulations and policies is an active subject of debate.^5^

In this case study, we explore the International Genetically Engineered Machine (iGEM) competition's experiences with gene drives, from rapid adoption of the technology by an undergraduate student team to development of oversight requirements within the competition. The nonprofit iGEM Foundation fosters open community collaboration and friendly competition through its annual synthetic biology competition.^6^ The development of iGEM's gene drives policy highlights the importance of governance (Box 1) and regulation, especially in the context of a rapidly evolving technological environment where formal regulations do not yet exist.^7^

**Box 1.** Working Definition of Governance^6,7^

Governance includes the norms, values, and rules of the processes through which public affairs are managed so as to ensure transparency, participation, inclusivity, and responsiveness. Governance also represents the structures and processes that are designed to ensure accountability, transparency, responsiveness, adherence to the rule of law, stability, equity and inclusiveness, empowerment, and broad-based participation.

## Gene Drive Project Surprised iGEM

iGEM's Safety and Security Committee (SSC) incorrectly assumed it would have several years to develop a policy on gene drives before a student team attempted to build one. In reality, however, it took only 11 months from a key publication demonstrating proof of principle in yeast before a related iGEM project was developed.^8,9^

iGEM evaluates and adjusts its policies annually in response to horizon scanning—early detection and assessment of emerging technologies—and new evidence on the policies' effectiveness.^10,11^ Despite having discussed the topic, the SSC had not yet developed a gene drive policy in 2016 when a team from the University of Minnesota attempted to build a drive as their project (Box 2). iGEM considers this to have been a near miss. The Minnesota team did not make a functional gene drive but managed to get some of the components to work. They were aware of potential environmental hazards from gene drives and took safety into account; for example, they proposed developing both a gene drive and a recovery drive to reverse any changes made. They also highlighted ethical and social issues surrounding use of this technology.

**Box 2.** Team Minnesota 2016 Project Abstract^9^

“Gene drives induce biased inheritance of specific genes and are currently being considered as a method of regulating the mosquito population; however, the ability of gene drives to spread quickly through entire populations raises ethical concerns, especially when the gene affects reproduction. In order to address this concern, we created both a gene drive and recovery drive, modeling the system in yeast. Our gene drive acts by removing the ADE2 gene from the yeast using the CRISPR/Cas system, causing the yeast to turn red, while the recovery drive replaces the ADE2 gene, allowing the yeast to return to its original color. The recovery drive is induced in the presence of tetracycline, allowing this recovery drive to be chemically induced as a safety mechanism, counteracting the work of the gene drive. This gene drive/recovery drive system could allow for regulated control of mosquito populations worldwide, the protection of information stored in DNA, and various other applications.”

iGEM did not realize the team was working on a gene drive until the team arrived to present their project at the end of the competition. The team was not working with pathogens, invasive species, or any other activities considered by iGEM to be especially risky and, therefore, subject to greater scrutiny. The team completed a general safety form about their project but it was not flagged by the third-party internationally certified biorisk management professional that reviewed it. It is likely that many such professionals were unaware of gene drives in June 2016. As with many of its policies (eg, on dual-use research of concern), iGEM also relies on institutional oversight of teams by their respective high schools, universities, commercial laboratories, etc. Ultimately, the SSC determined that the institutional biosafety office at the University of Minnesota had not established reporting requirements on gene drives and had not been informed that the team was working on a gene drive. The institutional biosafety office was also unlikely to have been familiar with gene drive hazards and risk management in 2016.

## iGEM's Response to a Gene Drive Project

iGEM responded to the team's gene drive project by engaging with the students, rapidly convening a group to work with them, and developing a policy to govern future gene drive-related projects.

The SSC spent considerable time with the team members exploring their work. From the outset, the team was eloquent and engaged in considering the broader implications of its project. While such engagement is unusual in many contexts, this has become the expectation in iGEM competitions. iGEM has a strong focus on human practices and expects teams to proactively engage with the social context of their work and responsibly address real-world problems.^12^ Before presenting their work, the team members were candid that they had not anticipated the extensive scrutiny their gene drive project received.

Members of the SSC convened and drafted an initial gene drive policy during the October 2016 iGEM Jamboree, which was publicly released in February 2017.^13^ Through a series of meetings, the committee created a policy that was reviewed by leading gene drive researchers and adopted within a matter of days. This quick work was possible because members of iGEM's SSC had already been heavily involved with complementary efforts to consider gene drives within the broader synthetic biology community, such as those within the Synthetic Biology Engineering Research Center,^14^ and because iGEM could draw on existing relationships with key researchers in the field and thought leaders who could provide oversight of the technology.

iGEM's SSC was not the only group interested in the student team's work. The project was reported more widely in the press, although an article published noted that leading gene drive researchers did not consider the project dangerous.^15^

The policy was updated 2 years later in May 2019, as part of iGEM's adaptive risk management approach, which includes periodic evaluation, adjustment, and review of policies that manage biological risks.^16^ In the revised policy, iGEM incorporated the practical experience of using the gene drive policy, which is an example of its experimentation in biosecurity governance.^17^ A timeline of gene drive-related policy and technological developments is presented in the Figure.

**Figure.**
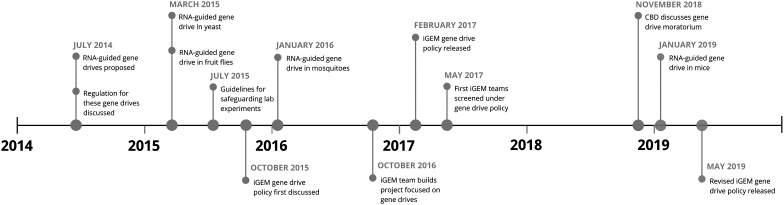
A timeline of gene drive-related policy and technological developments. Abbreviation: CBD, Convention on Biological Diversity.

## iGEM's Gene Drive Policy

Under the current gene drive policy (Box 3),^18^ iGEM teams cannot work on gene drives without a special exception from the SSC. For this policy to work in practice, iGEM developed a clear definition of a gene drive, determined how to monitor work with them, implemented a well-defined process for reviewing and approving research and ensuring adequate mentoring, and updated and revised its policy in light of technical and societal changes.

**Box 3.** iGEM's Gene Drive Policy (Updated 2019)^18^

Gene drives are not allowed in iGEM projects without a special exception from the Safety and Security Committee (SSC). Teams will need to convince the SSC that:
• There will be no environmental release – this is the existing iGEM policy for all projects and not just on gene drives.
• That the project is safe – iGEM has adopted an adaptive risk management approach for gene drives. The SSC will evaluate your project proposals with reference to host organism (chassis), modifications (including any associated parts) and containment measures. Teams should make use of the published framework for the risk assessment and management of gene drive technology in contained use.^19^
• Your team is implementing and adhering to the measures proposed by Akbari et al^20^ in “Safeguarding gene drive experiments in the laboratory.”
• Your team has notified the SSC that you are considering or planning to use gene drives in your project and you and your faculty advisor have participated in a mandatory conference call with experts on drives and on safeguards.
• Any orders for commercially produced genetic material placed by your team must be screened for regulated sequences.
• None of your parts submitted to the registry contain a functional gene drive – a drive in a single part will not be accepted and this can have implications for medal criteria.

### Deciding on a Definition of Gene Drives

Defining a gene drive is inseparable from decisions about how it should be governed.^5^ Decisions about iGEM's gene drive definition and governance system focused on 3 aspects of gene drives: their population-level potential effects, the rapid rate of advancement in the field, and the level of uncertainty on how they might be developed.

Since the aspect of concern related to gene drives was the potential impact on the wider ecosystem, rather than a particular technology, iGEM crafted a definition that focused on function (Box 4).^13^ iGEM developed a functional definition of gene drives to avoid becoming outdated in a rapidly developing technical area.^21^ After 2 years, iGEM reviewed the definition and included specific exemptions in response to teams incorrectly self-declaring their projects as gene drive-related (Box 3). The SSC finalized an updated definition of gene drives in November 2018, but the 2019 competition website would not be released for several months. Because teams regularly begin planning their next project as soon as a competition cycle is completed, the SSC updated the 2018 competition website with the revised definition after its adoption. As a result, the archived 2018 competition website shows the 2019 definition.

**Box 4.** iGEM's 2017 Definition of a Gene Drive^13^

“For the purposes of iGEM, a gene drive includes Cas9 (and other endonucleases, such as dCas9 and Cpf1) integrated into the genome (including through the use of gRNA) of a sexually reproducing eukaryotic organisms (including organisms that reproduce both sexually and asexually, such as yeast) and/or the use of a drive to impact the progeny.”

The incorrect self-declarations highlighted confusion as to what was covered by the policy and what was not, representing a limitation of the function-based definition of gene drives. iGEM therefore expanded and clarified certain terms, for example, noting that “bias the inheritance frequency in an organism's progeny” was not meant to include work in somatic cell lines that cannot reproduce sexually (Box 5).^18^ In the process of writing this case study, the authors realized that these expansions and clarifications could have been construed as exhaustive rather than indicative and, therefore, changed the definition to ensure that parts were “not limited to” those described.

**Box 5.** iGEM's 2019 Definition of a Gene Drive^18^

“The purpose of a gene drive is to **bias the inheritance frequency of a genetic marker in an organism's progeny**. For the purposes of iGEM, a gene drive includes (but not limited to) a gene or genes for recombinases or endonucleases (such as Cas, Cpf1, HEG, TALEN, ZFN) **site-specifically integrated** into the genome of a eukaryotic organism. These must be accompanied by a mechanism, such as a guide RNA (gRNA) in the case of Cas9, directing their site specificity if this is not already inherent in the protein sequence (as in the case of a ZFN). The recombinase or endonuclease used may be Cas9, Cas12a/Cpf1, or any other engineered or natural variant. All organisms capable of sexual reproduction are included. This also encompasses organisms such as yeast which reproduce mainly asexually (see exclusions below).
Exclusions:
• Genomic integration must be specific to the site targeted by the endonuclease, or a reasonable chance must exist for this to occur. Random insertion of the endonuclease (for example by lentiviral vector) does not constitute a gene drive.
• Work in somatic cell lines (i.e. non-germline) that are unable to reproduce sexually or evolve into the organism they are derived from is excluded. Examples of somatic cell lines include HEK293 and BV-2.”

The definition decision, like other governance decisions, also needed to address uncertainties related to the many ways that drive functions could be produced. This required both the definition and the governance approach to be adaptable in light of technical and societal advances.

Other groups have made different decisions about what a gene drive is and how it should be governed. Alphey et al^22^ collected many definitions from stakeholders, noting that “gene drive” is a term that may be used to describe “processes or phenomena, material objects, or intentions.” It is important to keep in mind the intention of a definition when considering its suitability for a particular governance system.

### Determining How to Monitor Work on Gene Drives

iGEM requires teams to self-declare any intention to use gene drives, and failure to self-declare is grounds for disqualification from the competition. iGEM is aware that many teams are either not aware of the existing rules or fail to follow them.^16,23^ As a result, iGEM developed a system with multiple reporting pathways and a multilayered system for detecting unreported research.

One way to improve self-declaration as a form of monitoring is to increase the likelihood that a team realizes they are undertaking work relevant to gene drives. To accomplish this, iGEM makes use of its:

- **Gene drive policy** – which directs the team to consult with the SSC on research related to gene drives.
- **White list** – which provides details of what needs prior approval for use in the competition, including experiments likely to bias the inheritance frequency of a genetic marker in an organism's progeny, such as a gene drive.
- **Safety and security form** – which must be completed by every team twice per competition cycle and requires teams to declare whether they are undertaking activities listed on the white list that need prior approval.

To increase the likelihood of detecting undeclared gene drive research in its competition, iGEM gives teams additional opportunities to describe their work, and reviews these descriptions for safety concerns:

- **Project description** – in addition to targeted safety questions, teams provide a general description of what they intend to accomplish on their safety and security form.
- **Project review** – all safety and security forms provided by teams are reviewed by internationally certified biorisk management professionals who look for gaps in the hazards identified by teams.
- **Wikis** – every team also writes up their project on a dedicated website that can be collaboratively edited by team members. The project wiki includes an overview of the project, details of research pursued, results achieved, and associated laboratory notes. Team wikis are reviewed as part of the safety and security review as well as a core part of competition judging.
- **Optional workshops** – all teams are invited to participate in events that are partly intended to identify projects that may pose an elevated risk, or that may require additional safety or security support from iGEM.

### Soliciting Expert Review and Ensuring Appropriate Mentorship

The comparatively high risk associated with gene drive research prompted iGEM to add a 2-step expert review process:

- **Internal review** – as with the use of any organisms, parts, or activities not covered by iGEM's white list, prior approval from the SSC is required. This specifically includes any gene drive-related research. Teams request permission by providing a more detailed risk assessment and management plan (Supplementary Material 1, www.liebertpub.com/doi/suppl/10.1089/hs.2021.0157). These plans are reviewed by iGEM's SSC, which includes experts from many different countries, with a variety of cultural backgrounds, technical expertise, and professional roles. The SSC must be persuaded that the planned work can be carried out safely, securely, and responsibly.
- **External consultations** – iGEM's gene drive policy requires all teams planning such work to participate in a mandatory conference call with experts on gene drives and safeguards (Box 3). At a minimum, these consultations include a principal investigator experienced in carrying out gene drive research and a current or former regulator who is familiar with gene drives and their unique properties. The experts then review the team's plans and protocols in more detail and help the team improve them. Any revised plans and recommendations from the experts are then considered by iGEM's SSC.

### Adapting Risk Management as Norms Emerge

In 2016, when the Minnesota team undertook its project, no national gene drive–specific policies or regulations existed that iGEM could use. Academic publications, however, included procedures and practices for safeguarding gene drive experiments in the laboratory.^20^ From the first iteration of its policy, iGEM required that teams make use of the measures described in the academic literature.

The 2019 review of the policy incorporated new guidance, such as practical frameworks for risk assessment^19^ and guidance on adopting an adaptive risk management approach.^24^ Since 2016, more countries have released national position papers and guidance on gene drives. Before granting permission to use a gene drive, iGEM requires teams from these countries to describe how they will comply with national rules and follow relevant local guidance.

In addition, iGEM shares with teams guidance on core commitments for field trials of gene drives, although it is outside the scope of iGEM's gene drive policy because it involves use beyond containment.^25^

### Enactment of the Policy to Date

Since the Minnesota team's 2016 project, gene drive research has not been conducted within the iGEM competition. Three teams in 2017 and another 3 teams in 2018 reported working on gene drive–related projects. Upon review by the SSC, it was determined that these teams were not pursuing gene drive research. In some cases, teams were randomly inserting an endonuclease into the genome (eg, by a lentiviral vector), rather than attempting site-specific integration. In other cases, the teams were targeting somatic cells and were therefore unlikely to have any impact on progeny. No team has self-declared a gene drive project since the definition was revised for the 2019 competition.

There may be several reasons why teams have not subsequently pursued gene drive projects for the competition,^18^ and reflecting on these reasons is a key part of learning from our experience in governance. First, iGEM's “no, but” policy sends a clear signal that positions gene drive policy between strict prohibitions, such as bans on human experimentation and environmental release, and more permissive policies such as “yes, but” policies on human subject-related research and antimicrobial resistance. Second, the policy sets a comparatively high bar for SSC approval of gene drive projects. The policy adds logistical and administrative burdens that are not required for other types of projects. Third, the policy directs attention to gene drives in the training and during early engagement with teams as they develop their projects. While this early engagement helps detect undeclared gene drive research, it may also dissuade teams from considering this research area.

## Lessons Learned for Governance of Emerging Technologies

iGEM's experience developing an adaptive governance system for gene drive projects highlight 10 general insights important for the governance of emerging technologies (Table). These insights include who should be involved, what the aims are, how to keep abreast of technological and societal changes, why greater transparency and honesty is important, where there are opportunities to build on existing efforts, and when action may be required.

**Table.** Insights for the Governance of Emerging Technologies Drawn from iGEM's Experiences with Gene Drives

Governance Insight	iGEM Experience
Involve a broad set of stakeholders in a technology, including those developing and planning to use it, those charged with managing relevant risks, and those who could be affected by it.	Before iGEM considers a gene drive project safe, secure, and responsible, the team must discuss its plans with both an experienced gene drive researcher and a current or former regulator familiar with gene drives. More broadly, competition requirements define success in iGEM to include careful reflection on how their work affects the world.
Collaborate with those already thinking about how to manage risks from new technologies.	No existing national regulations or formal guidance were in place when iGEM was confronted with a gene drive project, but other groups were already considering how risks may be managed. Engaging with these experts and processes notably enhanced iGEM's ability to act quickly and effectively.
Raise awareness of risks in relevant communities and have systems in place to identify relevant research.	Despite iteratively refined rules, tailored tools to support teams, and active outreach, some teams each year are still unfamiliar with iGEM's safety and security rules. iGEM has implemented a system with multiple reporting pathways and a multilayered system for detecting unreported research.
Create a continuous policy development and review process, including horizon scanning.	iGEM holds an annual workshop to identify issues it will need to address over the coming year, which feeds into revisions of its gene drive policy.
Increase transparency around near misses and community work to reduce risk of future events.	iGEM is sharing its own experience with a near miss in the hope of strengthening efforts to govern gene drives.
Think carefully about how we define what we are worried about.	iGEM chose to use a functional definition of gene drives to focus its concerns on potential population-level effects while also reflecting the level of uncertainty on how they might be developed.
Explore integration of different types of risk, including moving beyond a pathogen-centric model.	iGEM's failure to identify a gene drive project earlier was attributed to its narrow focus on traditional risks, such as safety risks caused by pathogens and containment risks posed by non-indigenous species.
Explicitly recognize uncertainty, both in our understanding of the technology and its implications, and adopt approaches to address it.	There are several ways a gene drive may be built and much of our understanding of their behavior outside the laboratory is based on modeling. When iGEM was presented with its first gene drive project, many safety technologies were only theoretical.
Experiment in governance and generate a strong evidence base. Try to show whether governance interventions are having the desired impact and how they might be improved.	iGEM has experimented with its gene drive definition, improving it through an iterative process based on real-world experience. iGEM has also collected data on interest in working with gene drives, which provided key insights to refine its policy.
Be ready to work quickly.	While iGEM expected to have several years to develop a policy, there were less than 11 months between a key publication on gene drives and an iGEM team presenting the results of a gene drive project.

When applied to the specific context of gene drives, the authors used these general insights to identify 6 key lessons that could be applied to US and other country efforts to develop relevant governance systems for gene drives. While noting that there are many differences between governance of gene drives in an international competition and governance and regulation at the national level, the authors believe that these insights may prove useful for those tasked with such regulation.

### 1. Gene drives deserve special attention because of their potential for widescale impact, and remaining uncertainty about how to evaluate intergenerational and transboundary risks

Relevant insights for the governance of emerging technologies:

- Think carefully about how we define what we are worried about.
- Increase transparency around near misses and community work to reduce risk of future events.

As discussed earlier, iGEM believes gene drive research poses a particular risk because of its potential to impact an entire ecosystem. Additionally, as iGEM's own experience shows, gene drives may be within the grasp of small groups of nonspecialists with comparatively few resources.

It is critical to better ascertain how to assess risks associated with gene drives. Gene drives can potentially have intergenerational effects, which complicates efforts to assess their potential to cause harm. Gene drives also have the potential for transboundary spread, meaning that attendant risks can spread across different communities and regulatory frameworks. This potential for spread highlights the need for common approaches to understanding and assessing risks from gene drives.

The continuing uncertainty on how best to assess the risks posed by gene drives is reminiscent of other science policy challenges. Past efforts have resulted in developing frameworks to better understand and characterize risks, such as expanding the “dual-use dilemma” through 7 experiments of concern—broad categories of research involving pathogens considered to pose particular risk of diversion to nonpeaceful purposes—which helped researchers identify what research was most likely to be relevant.^26^ iGEM adopted a similar approach, and through its white list has included gene drives as a type of research that is subject to more in-depth risk assessment and oversight.

### 2. An adaptive risk management approach is logical for gene drives because of the rapidly changing technological environment

Relevant insights for the governance of emerging technologies:

- Be ready to work quickly.
- Collaborate with those already thinking about how to manage risks from new technologies.

iGEM's experience shows that organizations must respond to gene drives more rapidly than the traditional timelines of regulation updates. iGEM thought it had more time to develop a policy for gene drive research, and creating an organizational policy is considerably simpler than at the national level. The United States should assume that developing and finalizing regulations will take time and should also prepare for relevant research taking place prior to those regulations being in place.

Based on iGEM's experiences, organizations should develop structures to govern gene drives in advance of more formal regulation. This means deliberately working to ensure that those involved in relevant work have a greater awareness of risks and are willing to invest time and effort into deliberately managing them. It is vital that different groups with a shared responsibility for governance revisit their responsibilities in light of changes in technology and society. This is where the practice of governance implementation, rather than theory, comes into its own. Testbeds like iGEM are critical for both experimentation in implementation and for learning lessons that can influence broader regulatory and policy decisions.^17^ A major challenge with such an adaptive risk management approach will be implementing a governance system that works for multiple, diverse communities. Unlike work with other hazards, the risk may not be confined to a well-defined community (eg, pathogens and infectious disease research) but could be from apparently unconnected research communities unintentionally making a gene drive.

### 3. Review by individual technical experts cannot scale and may fail to incorporate other forms of expertise, so must be complemented with a range of alternative governance methods

Relevant insights for the governance of emerging technologies:

- Experiment in governance and generate a strong evidence base. Try to show whether governance interventions are having the desired impact and how they might be improved.

iGEM has been fortunate to consult with experts in the science of gene drives, such as George Church and Kevin Esvelt—both of whom had built gene drives and published their findings before the 2016 Minnesota team's iGEM project.^1^ There are a limited number of gene drive experts, however, so governance based purely on consultation may not scale or be suitable for general policy development. Equally, relying on the views and insights of a single individual (or very small group) carries some risks—for example, what happens if they get something wrong?

As the United States considers governance options and regulatory oversight of gene drive research, it should prepare to operate in research communities that lack the expertise needed to understand its risks and benefits. Safe, secure, and responsible gene drive work in such communities requires the development of better tools and technologies to assess and manage risk, and a more systematic approach to learning lessons from the limited dataset available.^21^ The United States will need to carefully consider any attempts to expand the key gene drive expert community and whether such efforts may increase the likelihood of gene drive research taking place, thereby increasing the risk of harm. Furthermore, the United States may consider changing how expert input is integrated into governance systems—perhaps exploring how the “wisdom of crowds,” machine learning, or other approaches may be used to triage the need for expert input. The authors encourage others to experiment with these alternative governance mechanisms and share lessons learned.

### 4. Gene drives have already demonstrated their ability to challenge governance systems, and current laboratory biosafety and biosecurity review may not capture gene drive research or its components in practice even if they are covered theoretically

Relevant insights for the governance of emerging technologies:

- Raise awareness of risks in relevant communities and have systems in place to identify relevant research.
- Explore integration of different types of risk, including moving beyond a pathogen-centric model.

iGEM failed to identify that a team was working on a gene drive for several reasons. Some of these reasons were that the team in question did not use (1) select agents, (2) anything covered by export controls, (3) parts from pathogens, (4) any of the 7 experiments of concern, (5) exotic plants or animals, or (6) exotic microbes or traditional invasive organisms. If any of these had been required for gene drives, the team would have been flagged by iGEM's project screening.

The major challenge for the United States as it considers how to govern gene drives is that its potential risks are different from other technologies that have more established governance systems. Gene drives do not involve pathogens or toxins and therefore may be missed by existing biosafety or biosecurity rules. In addition, the potential impact from an accidental release of a gene drive should be considered greater than other hazards that are traditionally associated with failures in laboratory containment.

### 5. In the context of uncertainty around gene drives, risk management for research and development must incorporate discussions of values and broader implications of the work

Relevant insights for the governance of emerging technologies:

- Explicitly recognize uncertainty, both in our understanding of the technology and its implications, and adopt approaches to address it.
- Involve a broad set of stakeholders in a technology, including those developing and planning to use it, those charged with managing relevant risks, and those who could be affected by it.

iGEM believes:

*Decisions in science and engineering shape, and are shaped by, the societies we create. Social, political, economic, and ethical aspects of synthetic biology cannot be an afterthought of research and development. Rather, they should be considered from project conception all the way through the innovation process. Synthetic biology is not just science or engineering. It is a practice of building life and the societies and environments that support that life.*^27^

iGEM considers the values and broader implications of research, which is captured as teams engage with the question of human practices, by thinking deeply and creatively about whether a synthetic biology project is responsible and good for the world. Teams are encouraged, supported, and rewarded for considering how their work affects the world, and how the world affects their work.

As the United States develops the process it will use to govern gene drives, inclusivity will be key. Deliberations about safe, secure, and responsible development and use of gene drives will have to go beyond technology. Those who pursue and govern gene drive research will need to engage with and consider the views of those who might be impacted by the use of gene drives. These considerations should be incorporated into the governance system and any regulations developed in the future. Similar efforts have already been proposed for field trials involving gene drives.^25^

### 6. A regular technology horizon scanning capacity is needed for the early identification of advances that could pose governance system challenges

Relevant insights for the governance of emerging technologies (Table):

- Create a continuous policy development and review process, including horizon scanning.

Although it had not yet developed policies related to gene drive research, iGEM had successfully identified gene drives as an issue to watch before the Minnesota team introduced its project in 2016. An initial round of discussions on gene drives and their implications took place in September 2015 at iGEM's annual safety and security meeting. These meetings involve iGEM's own safety and security community, as well as partners within synthetic biology and oversight of technology more broadly. Because of these meetings and discussions, the SSC was well placed to develop a gene drive policy rapidly: core issues had been identified and discussed, members of the community were comfortable with the technology, and potential partners had been identified for more in-depth assessment.

## Conclusion

It is noteworthy that the United States is only now considering a national oversight policy. The country needs a standardized, forward-leaning mechanism to identify emerging technologies, such as gene drives, that could present regulatory and oversight challenges. Identifying relevant advances early on would enable the development of better regulations and stronger governance. Such a mechanism should be multidisciplinary and have a wider scope than focusing solely on gene drives or synthetic biology. It needs to bring together expertise across a wide range of domains and relate to regulatory regimes housed within diverse government departments and agencies. Ideally, the United States could expand existing security horizon-scanning activities undertaken by the Office of Science and Technology Policy within the White House to consider a wider range of biological risks.^28^ Other countries should likewise consider what approaches can enable the types of proactive and adaptive policies needed to safeguard a rapidly advancing field.

## Supplementary Material

Supplemental data
