# Supplementary material for: iGEM and Gene Drives: A Case Study for Governance
Source: Health Secur. 2022 Feb 15;20(1):26–34. doi: 10.1089/hs.2021.0157 (PMC8892970; doi:10.1089/hs.2021.0157)
Supplement: Supplemental data [file Suppl_Data.zip › 21-0157 R1 Millett Gene Dr SF Figure title caption TE KF.docx]

Figure. A timeline of gene drive-related policy and technological developments. Abbreviation: CBD, Convention on Biological Diversity.
