# Supplementary material for: iGEM and Gene Drives: A Case Study for Governance
Source: Health Secur. 2022 Feb 15;20(1):26–34. doi: 10.1089/hs.2021.0157 (PMC8892970; doi:10.1089/hs.2021.0157)
Supplement: Supplemental data [file Suppl_Data.zip › 21-0157 R1 Millett Gene Dr SF Supplemental material 1.pdf]

iGEM 2021 Safety and Security Check-In Form

There are no deadlines for this form. It can be submitted at any time.

You should submit a Check-In before you acquire or use certain materials in your lab or before you undertake certain types of activities. Specifically, you should send us a Check-In for any organism, part, or activity that is not on the **White List**. We expect to reply to most Check-Ins within a few days. If you have not heard back from us in a week, please email *safety [AT] igem [DOT] org*.

Check-Ins are a way for you to quickly and easily ask iGEM's safety experts to review your plans for safely acquiring and using a higher risk organism/part/activity, and to approve your plans or suggest changes.

We encourage STUDENTS, instead of instructors, to complete this form. Please provide as much detail as possible.

Once the iGEM Safety and Security Committee has approved your Check-In by email, you may begin working with the material (organism or part) or activity. You may Check-In for as many organisms/parts as you wish, and any team member may send a Check-In at any time until the Jamboree.

Frequently Asked Questions about Check-Ins

What if I am unsure whether my organism/part requires a Check-In?

Ask us! Email *safety [AT] igem [DOT] org* to ask questions about what requires a Check-In. Or, because the Check-In form is short and easy, you can submit a Check-In even if you are unsure whether it is necessary.

We want to do a little preliminary work with an organism/part, but we might not use it for our final project. Do we still have to Check-In before we acquire the organism/part?

Yes! Please Check-In for every organism/part you want to acquire that is not on the White List, even if you will not use it in your final project. You can tell us in the "Further Comments" section that it is not for your final project, or that you are unsure. We have made the Check-In form short and easy, so you can Check-In for many parts, even ones you are not sure about using.

Uh-oh! We misunderstood the White List, and we already started working with an organism/part that requires a Check-In. What do we do?

Just let us know. Email *safety [AT] igem [DOT] org* to describe the situation, and send us a Check-In promptly. Tell us in the "Further Comments" section what you have done with the organism/part already.

We are going to use a lot of parts. May we combine them on a single Check-In?

If the parts all come from the same parent organism, you may combine them on a single Check-In, but make sure you give complete information about each part. If the parts come from different parent organisms, please send separate Check-Ins.

You approved our Check-In for an organism/part. Do we still need to ask our university/institution about it? What about checking local laws?

Yes! The iGEM Safety Committee does not replace institutional review boards, or your local government. You are responsible for obtaining from your university or government any approvals that might be necessary. Your university or government might have different rules about what organisms/parts require special approval.

We are only using organisms/parts from the White List, and therefore we do not need to submit any Check-Ins. Do we still need to ask our university/institution about our project? What about checking local laws?

Yes! Again, the iGEM Safety Committee does not replace institutional review boards, or your local government. Even if you are only using organisms/parts that are generally considered "safe", you still have the responsibility to follow good laboratory procedures. Also, you are responsible for ensuring that your project complies with the rules of your university/institution, and with the laws of your nation.

Safety Check-In Form

Team Selection

Please choose a team

Choose team...

1. Check In Information

A- Choose an existing Check-In:

(New...)

B- Start a new Check-In:

Check In name:

Please name your form to help identify what organism, part or activity you are checking in) If this Check-In is for a part: Enter its function. Please do not name it after either the organism that the part originally comes from, or the name of the chassis organism that you will put the part into.

This Check-In is for a:

- A. Whole Organism
- B. Part
- C. Activity

2. Contact Information

3.A Organism Information

3.A.1 What Risk Group is this organism? [Help about Risk Groups]

- Risk Group 1
- Risk Group 2
- Risk Group 3
- Risk Group 4
- This organism is not a microbe. It does not have a Risk Group.
- Other *(please describe):*
- Unknown *(please comment):*
- N/A

Alert

iGEM teams should not use Risk Group 3 or 4 organisms, and they should not work in Safety Level 3 or 4 labs. If you are planning to work at Safety Level 3 or 4, contact *safety [AT] igem [DOT] org* right away!

3.A.2 Is the organism on the Australia Group List, or on the U.S. Select Agents and Toxins List or national equivalents?

*If Yes, please email safety [AT] igem [DOT] org. These organisms and their parts are restricted for international shipment. See **Safe Shipment** for more information.*

- Yes
- No
- N/A

3.A.3 What risks have you identified from the use of this organism?
Please provide relevant links to supporting information, such culture collection data sheets

---

3.A.4 What measures have you put in place to manage the risks you have identified?

3.A.5 How will you acquire this organism?

- ○ My institution *(supervisor, another lab, central store, etc.)*
- ○ Isolating from an environmental medium *(e.g. soil, water)*
- ○ Ordered from a supply source *(such as a culture collection, repository, or organism design company - please indicate which company)*
- ○ N/A

3.A.6 Further Comments
Anything else you want to tell us about this organism and how you will use it

3.B Part Information

3.B.1 What is the parent organism for this part?

- Organism name *(please provide as much detail as possible)*
- No natural comparator *(including parts designed in silico)*
- N/A

3.B.2 What is the natural function of this part in its parent organism?
(If this is not a part taken from nature, please note this)

3.B.3 Is the part toxic to humans?
For example, a protein toxin such as Botox (Botulinum toxin), or if it is an enzyme that synthesizes a toxic small molecule.

- Yes *(Please provide details)*
- No
- N/A

3.B.4 Is the part a virulence factor, does it mimic one, or could it reasonably be expected to act as one in the chassis you are planning to use? [Help about Virulence Factors]

- Yes *(Please provide details)*
- No
- N/A

3.B.5 Does the part, by itself, pose any risks to you, your colleagues, communities, or to the environment if it got out?
For example, a protein toxin that could affect indigenous plants or animals, or if it synthesizes a toxic small molecule, or encodes antimicrobial resistance.

- ○ Yes *(Please provide details)*

- ○ No
- ○ N/A

3.B.6 Have you altered the part from its natural sequence, structure or function?

- ○ Yes *(Please provide details, included intended changes to structure or function)*

- ○ No
- ○ N/A

3.B.7 Does the part, when combined with other parts or when used in your chassis, pose any risks to you, your colleagues, communities, or the environment if it got out?

- ○ Yes *(What are the risks? Please provide details)*

- ○ No
- ○ N/A

3.B.8 In what chassis will you use this part?
(Please provide details of the organism or cell-free system in which this part will be used)

3.B.9 For what will you use this part?
How does it fit into the overall function of your project?

3.B.10 How will you acquire this part?

- My institution *(supervisor, another lab, central store, etc.)*
- Isolating from an organism *(e.g. by PCR)*
- Ordered from a gene synthesis company (if so, please indicate which company)
- Ordered from another company *(such as a culture collection, repository, or organism design company - please indicate which company)*
- N/A

3.B.11 What measures have you put in place to manage the risks you have identified in Q3.B.5 or Q3.B.7?
(e.g. handling it in a separate lab area, wearing additional protective equipment)

3.B.12 Further Comments
Anything else you want to tell us about this part and how you will use it

3.C Activities

3.C.1 Which activity are you checking in?

- ○ Experiments likely to bias the inheritance frequency of a genetic marker in an organism's progeny, such as through the creation of a gene drive.
- ○ Experiments likely to render a vaccine ineffective.
- ○ Experiments likely to confer resistance to the World Health Organization's list of **Critically Important Antimicrobials** ↗.
- ○ Experiments likely to make hazardous biological agents more hazardous, such as enhancing the virulence or transmissibility of a human, plant, or animal pathogen, or altering its host-range.
- ○ Experiments likely to result in a novel hazardous biological agent, such as by rendering a nonpathogen virulent, or conferring degradation of damaging important materials *(such as electronics, plastics, etc.).*
- ○ Experiments likely to enable a pathogen or organisms capable of damaging important materials to evade common diagnostic or detection tools.
- ○ Experiments likely to make a biological agent or toxin more suitable for use as a weapon.
- ○ Another activity *(please describe)*
- ○ N/A

3.C.2 Why do you want to carry out this activity?
What is your rationale for needing to do it?

3.C.3 Please describe the planned protocol in as much detail as possible

---

3.C.4 What risks have you identified from this activity?

3.C.5 What measures have you put in place to manage the risks you have identified?

4. Further Comments

Anything else you want to tell us about the organism, part or activity described on this form? Any more details about risks from your project and how you are managing them? Or any ideas how we might improve how we manage risks covered by this form?

6. Sign off

Finished?

SUBMIT

Need to make changes?

UNSUBMIT

Want to erase this Check-In?

DELETE
